# Dichloroacetate, a selective mitochondria-targeting drug for oral squamous cell carcinoma: a metabolic perspective of treatment

**DOI:** 10.18632/oncotarget.2721

**Published:** 2014-12-08

**Authors:** Vitalba Ruggieri, Francesca Agriesti, Rosella Scrima, Ilaria Laurenzana, Donatella Perrone, Tiziana Tataranni, Carmela Mazzoccoli, Lorenzo Lo Muzio, Nazzareno Capitanio, Claudia Piccoli

**Affiliations:** ^1^ Laboratory of Pre-Clinical and Translational Research, IRCCS, CROB, Rionero in Vulture, Potenza, Italy; ^2^ Department of Clinical and Experimental Medicine, University of Foggia, Foggia, Italy

**Keywords:** oral squamous cell carcinoma, dichloroacetate, oxidative metabolism, mitochondria, reactive oxygen species

## Abstract

Reprogramming of metabolism is a well-established property of cancer cells that is receiving growing attention as potential therapeutic target. Oral squamous cell carcinomas (OSCC) are aggressive and drugs-resistant human tumours displaying wide metabolic heterogeneity depending on their malignant genotype and stage of development. Dichloroacetate (DCA) is a specific inhibitor of the PDH-regulator PDK proved to foster mitochondrial oxidation of pyruvate. In this study we tested comparatively the effects of DCA on three different OSCC-derived cell lines, HSC-2, HSC-3, PE15. Characterization of the three cell lines unveiled for HSC-2 and HSC-3 a glycolysis-reliant metabolism whereas PE15 accomplished an efficient mitochondrial oxidative phosphorylation. DCA treatment of the three OSCC cell lines, at pharmacological concentrations, resulted in stimulation of the respiratory activity and caused a remarkably distinctive pro-apoptotic/cytostatic effect on HSC-2 and HSC-3. This was accompanied with a large remodeling of the mitochondrial network, never documented before, leading to organelle fragmentation and with enhanced production of reactive oxygen species. The data here presented indicate that the therapeutic efficacy of DCA may depend on the specific metabolic profile adopted by the cancer cells with those exhibiting a deficient mitochondrial oxidative phosphorylation resulting more sensitive to the drug treatment.

## INTRODUCTION

Oral squamous cell carcinoma (OSCC) is the eighth most frequent neoplasm worldwide, with over 300.000 new cases reported annually [1]. Environmental hints, chronic inflammation and viral pathogens, as well as accumulation of multiple genetic alterations and epigenetic gene deregulation are possible etiopathogenic factors [2–4]. Despite the advances of screening and early diagnosis, the 5-years disease free survival in OSCC patients is still poor, principally due to the aggressive biology and refractariety of this tumour to conventional therapies. The lack of an adequate understanding of the cell biology of oral oncogenesis requires further efforts in the field of prognostic and predictive biomarkers for existing therapy as well as in the research of innovative molecular targets for anti-cancer therapy [4, 5]. Interestingly, ^1^H NMR-based metabolic profiling of different human OSCC cell lines recently delineated a significant deregulation of various metabolic pathways, including glycolysis [6]. Altered glucose metabolism is a common property of invasive cancer cells that typically show increased glycolysis and reduced mitochondrial oxidation regardless of the availability of oxygen. This metabolic reprogramming of transformed cells, known as Warburg effect, constitutes an enormous advantage for tumour growth, also contributing to the invasive/adaptive properties of cancer cells and to resistance to common anticancer agents. Even if the underlying biochemical and molecular mechanisms of this phenomenon are extremely complex and poor understood, this metabolic abnormality may be exploited as biochemical rationale to design selective therapeutic treatment [7–10]. Pharmacological inhibition of the Warburg effect with the pyruvate mimetic dichloroacetate (DCA) has provided impressive results in a wide spectrum of human cancers cells including pancreatic [11], colon [12], breast [13], ovarian [14], endometrial [15] neuroblastoma [16], glioblastoma [17] and T-cell lymphoma cells [18]. DCA shifts cell metabolism from glycolysis to mitochondrial glucose oxidation by inhibition of pyruvate dehydrogenase kinase (PDK), the selective inhibitor of pyruvate dehydrogenase (PDH) converting cytosolic pyruvate to mitochondrial acetyl-CoA, the substrate for the Krebs cycle. The ensuing reprogramming of the abnormal bioenergetic pattern in cancer cells proved to induce selective citotoxicity thereof [19]. Although glucose metabolism is a pivotal factor in the progression of OSCC and the glycolytic phenotype represents a significant negative biomarker of prognosis and overall survival in OSCC patients [20], nevertheless, to our knowledge, no experimental studies explicitly focused on the correlation between the metabolic profile of OSCC cells and on the possible effects of metabolic drugs. Since the cellular heterogeneity and the metabolic adaptation of tumours make the development of effective therapies challenging, in this study we characterized the metabolic phenotype of different OSCC-derived cell lines showing, for the first time, a straight correlation between the mitochondrial activity and the DCA sensitivity. This could represent one of the major pre-clinical steps for the use of selective metabolic drugs.

## RESULTS

HSC-2 and HSC-3 are human tumour cell lines established from metastasised lymph nodes of a gingival carcinoma with an invasive tendency more prominent in HSC-2. Both exhibit a missense mutation of the tumour suppressor p53 at codon 280^Arg/Thr^ and codon 176^Cys/Phe^ respectively, and low mtDNA repair ability [21]. The mutations in p53 of HSC-2/3 affect the expression of the tumour suppressor at the post-transcriptional level. The PE15 cell line, originated from a human dedifferentiated OSCC of the tongue, displays epithelial characteristics but, unlike HSC-2/3, expresses normal p53 (confirmed by Western blotting in this study - data not shown).

### Characterization of the mitochondrial OxPhos activity in OSCC cell lines

**Figure 1:**
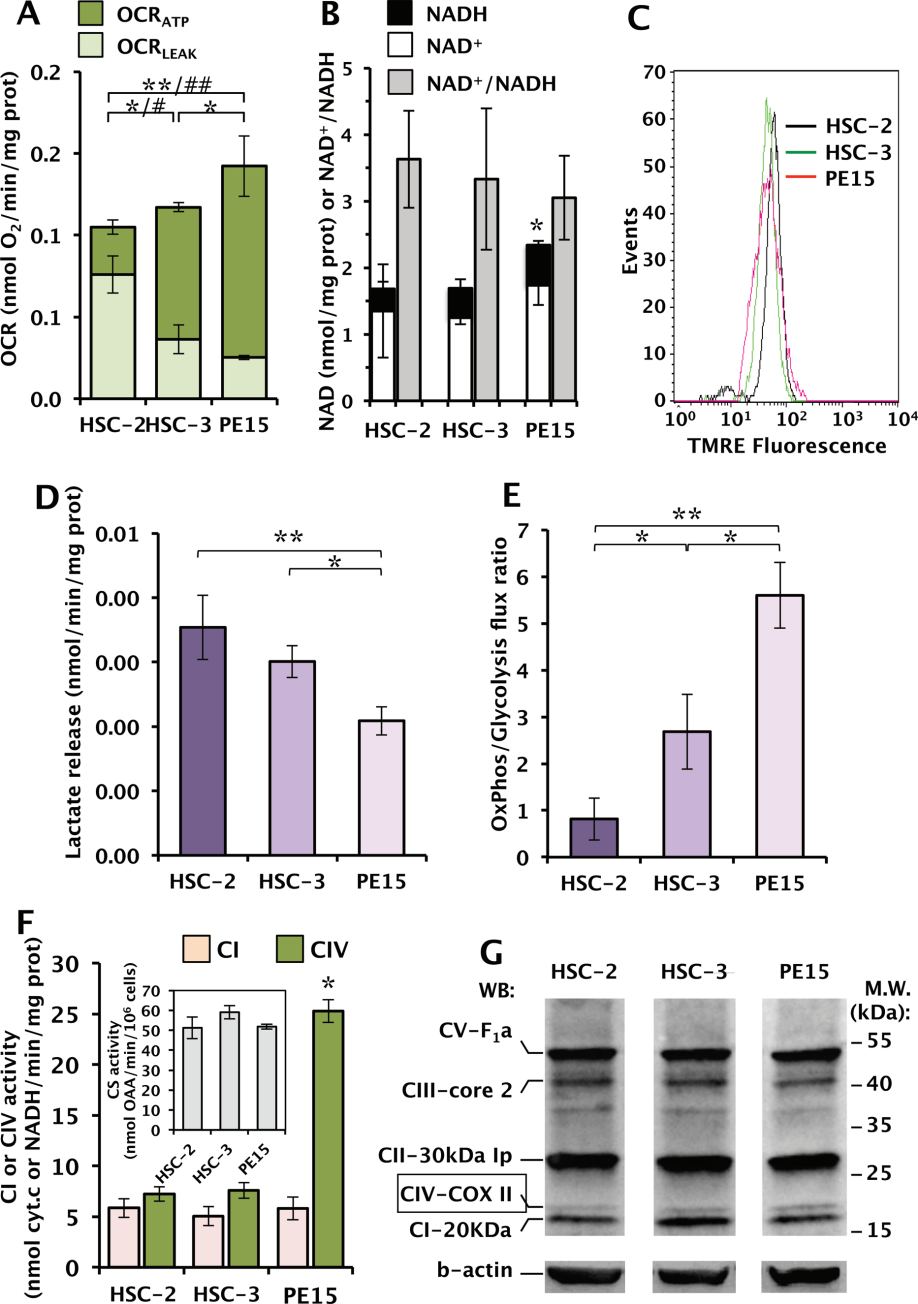
Comparative analysis of the metabolic profiles of different OSSC-derived cell lines **(A)** Graphical representation of OCR values in intact cells measured by respirometric assay: light green columns represent the OCR_LEAK_ values, obtained after addition of the FoF1-ATP synthase inhibitor oligomycin to intact cells; dark green columns indicate the OCR_ATP_ values calculated as the difference between the overall endogenous OCR and the oligomycin-insensitive OCR. The values reported are mean (± SEM) of six independent experiments with the OCRs normalized to the protein content. Statistical significance: (*) *P* < 0.05, (**) *P* < 0.01 for OCR_LEAK_; (^#^), *P* < 0.05, (^##^) *P* < 0.01 for OCR_ATP_. **(B)** Cellular content of NAD^+^ (empty columns), NADH (black columns) and of their ratio (grey columns) normalized to the protein content of each sample, calculated from three independent experiments. (*) *P* < 0.05 as total NAD content. **(C)** Flow cytometric analysis of ΔΨ_m_ in OSCC stained with the specific probe TMRE; 10,000 events for each sample were acquired and analyzed with the CellQuest software. **(D)** Measurement of lactate in culture medium; 2 × 10^6^ cells were plated and, after 24 h of incubation, the lactate released were determined as indicated in Material and Methods and normalized to the cellular proteins. The data reported means (±SEM) of three independent experiments. (*) *P* < 0.05, (**) *P* < 0.01. **(E)** Analysis of the OxPhos/Glycolysis metabolic flux ratio calculated as the ratio between the OCR_ATP_ (see panel A) and the lactate amounts (see panel D). Statistical significance, (*) *P* < 0.05, (**) *P* < 0.005. **(F)** NADH dehydrogenase (CI) and cytochrome c oxidase (CIV) enzymatic activities measured spectrophotometrically as detailed in Materials and Methods; the results are means (± SEM) of three independent experiments, (*), *P* < 0.05. The inset shows the citrate synthase (CS) activity measured on the same samples. **(G)** Protein expression levels of the five OxPhos complexes (CI to CV), determined by immunoblot assay on total cell lysates using a cocktail of specific antibodies; β-actin was used as loading control. The blotting is representative of three independent experiments.

Figure 1A shows the results of a comparative analysis of the rates of endogenous respiration in intact OSCC cells performed by high-performance oxymetry. The oxygen consumption rates (OCRs) were normalized to the cellular protein content and corrected for the residual oxygen consumption after the addition of the Complex I inhibitor rotenone, and therefore attributable to the mitochondrial respiratory chain activity. The HSC-2 cell line displayed the lowest OCR when compared to the HSC-3 and PE15 cell lines. In the presence of the FoF1-ATP synthase inhibitor oligomycin, the endogenous OCR was depressed but at different extent in the three OSCC samples (lighter colored columns in Fig. 1A). The difference between the overall endogenous OCR and that in the presence of oligomycin (darker colored columns in Fig. 1A) provides a measure of the oxygen consumption linked to the oxidative phosphorylation (OxPhos) (OCR_ATP_) and indicate a different OxPhos efficiency among the three OSCC cell lines in the sequence HSC-2<HSC-3<PE15. The oligomycin-insensitive OCR is a measure of the respiratory chain activity not linked to the synthesis of ATP and controlled by dissipative proton fluxes across the membrane also known as proton leaks (OCR_LEAK_). To notice, the residual OCR_LEAK_ displayed a 2–3 fold larger value in HSC-2 as compared with HSC-3 and P15 and the lowest OCR_ATP_.

Consistently, the cellular content of NAD, which regulates the oxidative metabolism (with mitochondria segregating the major intracellular NAD pool), was significantly higher in PE15 with respect to the HCS-2/3 cells (Fig. 1B). Conversely the NAD+/NADH ratio did not show significant differences in the three cell lines.

Since the proton motive activity of the mitochondrial electron transport chain is coupled to generation of a mitochondrial membrane potential (ΔΨ_m_), we measured it by flow cytometry using the specific probe TMRE. Surprisingly, the three OSCC cell lines, irrespective of the observed differences in the respiratory capacity, did not show significant differences in the uptake of the ΔΨ_m_-sensitive fluorescent probe (Fig. 1C). However, it has to be considered that i) the ΔΨ_m_ can also be partly generated by the reverse ATP-ase activity of the F1Fo-ATP synthase utilizing glycolytic ATP and ii) that OCR and ΔΨ_m_ are not linearly correlated.

The difference in the reported OxPhos efficiency may reflect a specific bioenergetic adaptation of the HSC-2 cell line in which, despite normal oxygen conditions, metabolism is more dependent on glycolysis, as described in the Warburg effect [8]. Consistently, the measured flux of lactate released in the medium was the highest in HSC-2 (+20% and +70% vs HSC-3 and PE15 respectively) (Fig. 1D) and consequently the ratio between the OCR_ATP_ and the lactate released (that can be taken as an indirect measure of the OxPhos/Glycolysis metabolic flux) was markedly reduced in HSC-2 as compared with the PE15 cell line with the HSC-3 resulting in an intermediate value (Fig. 1E).

The endogenous mitochondrial respiratory activity in intact cells is mainly controlled by the cytochrome c oxidase (complex IV, CIV), depending on the prevailing conditions [22]. The specific enzymatic activity of CIV was measured and as shown in Fig. 1F resulted to be significantly higher in PE15. The observed differences held also when normalized to the citrate synthase activity, which is used as an indicator of the cellular mitochondrial mass (see inset of Fig. 1F). Conversely, measurement of complex I activity did not result in major differences among the three OSCC cell lines. Assessment of the mitochondrial OxPhos complexes (CI to CV) using a cocktail of specific antibodies did not result in major differences in the expression of any of the respiratory chain complexes neither of the F_1_F_o_-ATP synthase with the exception of the CIV-marker subunit II, which resulted in a roughly 2-fold more intense immune-stained band in PE15 (Fig. 1G and densitometric analysis not shown).

### Effect of DCA on the phosphorylation state of the pyruvate dehydrogenase

**Figure 2:**
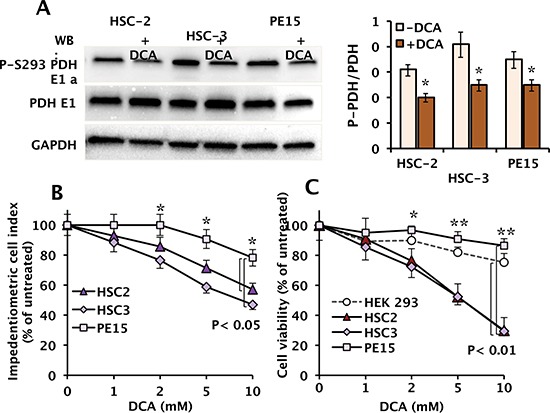
Effect of DCA on phosphorylation of the PDH-E1α subunit and cell growth and viability in OSCC **(A)** Western blot of the phospho-S293 E1α PDH and of the total enzyme subunit in OSCC cells following a 24 h incubation with vehicle and 10 mM DCA; GAPDH levels were used as loading control. The blot shown is representative of three independent experiments. Panel on the right: quantitative analysis of phospho-S293 E1 α PDH expression relative to total PDH E1α normalized to GAPDH, carried out by a densitometric analysis (“Image Lab” software, Biorad). The results are the means (± SEM) of three independent experiments. (*) *P* < 0.05. **(B)** Dose-dependent effect of DCA treatment on cell growth analysed by using an impedentiometric technique (xCELLigence RTCA MP System, Roche, Germany). Continuous monitoring of cell adhesion and proliferation was carried out for 24 h after incubation of cells with the indicated concentrations of DCA, and expressed as the percentage (%) of the cell index of untreated cells; means (± SEM) of three repeats. (*): *P* < 0.05. **(C)** Dose-dependence effect of DCA treatment on cell viability. Cells were exposed for 24 h to the indicated concentrations of DCA and viability determined with the MTS assay. Non-malignant human HEK 293 cells were used as representative of a non tumor cell line. Cell viability is expressed as the percentage (%) of untreated cells. The data shown are means (± SEM) of 6 independent experiments; (*) *P* < 0.05; (**) *P* < 0.01.

Before evaluating the effect of DCA on vital parameters of the OSCC cell lines, the efficacy of the compound to inhibit its recognized target pyruvate dehydrogenase kinase (PDK) was assessed by Western blotting using a specifici antibody recognizing the phosphorylation state of the E1α subunit (residue S293) of the PDH complex [23]. Figure 2A shows that the basal level of both PDH and P-PDH-E1 were comparable among the three OSCC cell lines and that following a 24 h-treatment with 10 mM DCA (an effective concentration reported in literature [13–17]) a significant reduction in the normalized level of P-PDH-E1 was observed with this value resulting slightly lower in PE15 as compared with HSC-2 and HSC-3 (45–50% vs. 20% inhibition).

### Effect of DCA on the cell growth and viability

Figure 2B shows the dose dependence of DCA on the growth-rate of the OSCC cell lines measured by an impedentiometric technique. The results obtained show that the cell index of the OSCC cell lines was drug-sensitive with HSC-2 and HSC-3 significantly more depressed by DCA as compared with PE15. The effect of DCA was then tested on the cell viability by the MTS assay confirming for HSC-2 and HSC-3 an even more pronounced distinctive sensitivity to the drug (Fig. 2C). Indeed at 10 mM DCA the viability of both HSC-2 and HSC-3 was around 30% whereas that of PE15 was 75–80% as compared to that of the representative non tumor cell line HEK 293 (human embryonic kidney cells).

### Effect of DCA treatment on mitochondrial respiration and lactate production

**Figure 3:**
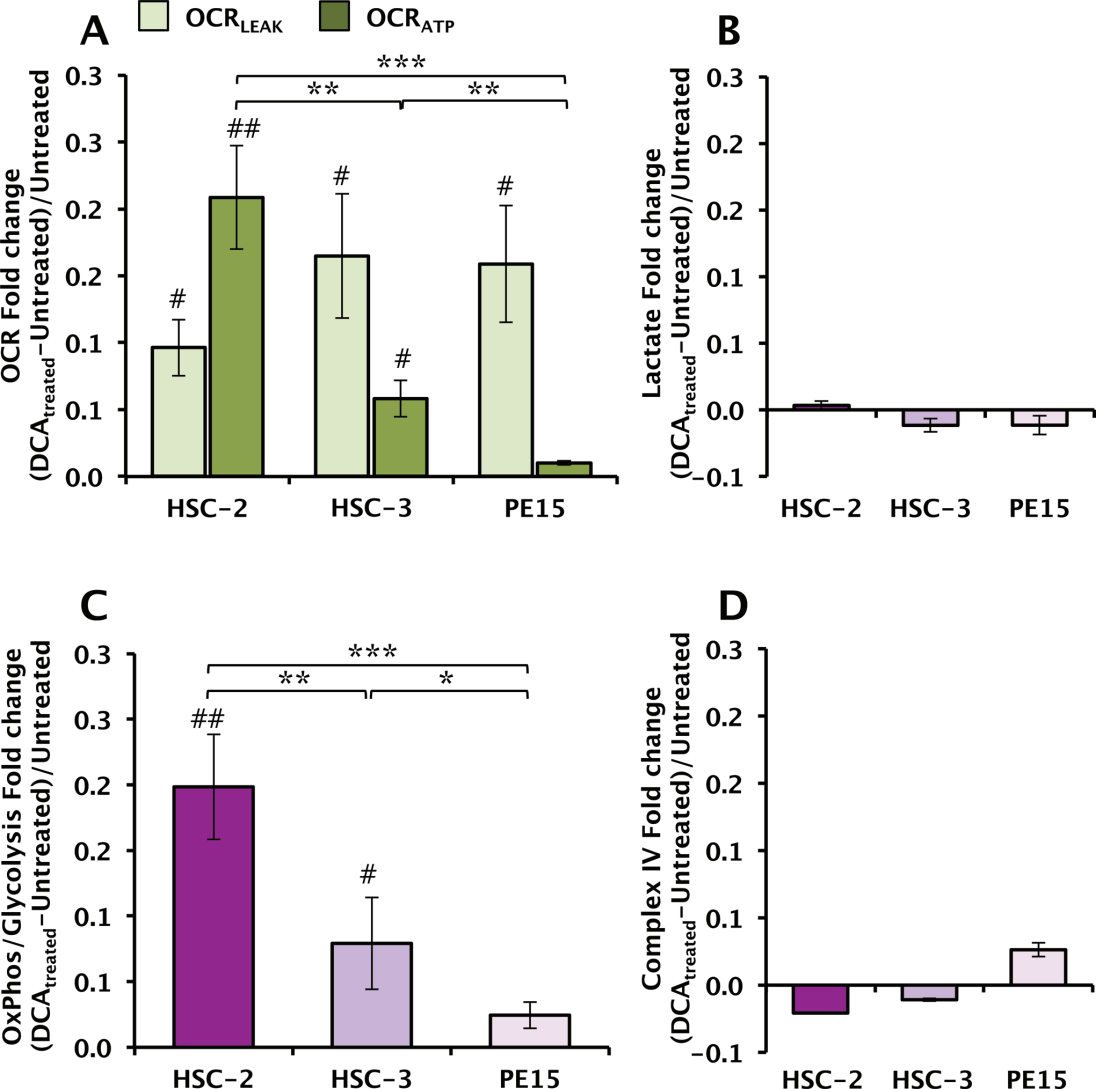
Effect of DCA treatment on the metabolic profile of OSCC cells **(A)** Endogenous respiratory activities; light and dark blue bars show the OCR_LEAK_ and the OCR_ATP_ respectively measured following exposure of OSCC cells to 4 mM DCA for 24 h. The values reported are fold-change calculated as the ratio of the OCR values in DCA-treated versus untreated cells and are the mean (± SEM) of three independent experiments. (**) *P* < 0.005, (***) *P* < 0.001 (#) *P* < 0.05 vs untreated. **(B)** Fold-changes of lactate release after 24 h-treatment with DCA; means (±SEM) of three independent experiments. **(C)** Fold-changes of the OxPhos/Glycolysis metabolic flux ratio (see Fig. 1E for explanation) in DCA-treated OSCC cells; means (±SEM) of three independent preparations assayed in parallel for OCR_ATP_ and lactate. (*) *P* < 0.05, (**) *P* < 0.01, (***) *P* < 0.005; (#) *P* <0.05, (##) *P* <0.01 vs untreated. **(D)** Fold-changes of the CIV activity in OSCC cells following DCA-treatment. The data reported are the mean (±SEM) of three independent experiments.

Activation of PDH is expected to enhance the metabolic flux through the tricarboxylic cycle fueling the mitochondrial respiration [23]. Figure 3A shows that 4 mM DCA - a mildly cytotoxic concentration - caused, indeed, an increase of the resting endogenous OCR in all the three OSCC cell lines. This was partly contributed by enhanced OCR_LEAK_ (light colored bars) and partly by enhanced OCR_ATP_ (dark colored bars). However, the relative largest effect of DCA was observed for the OCR_ATP_ in HSC-2 while that on PE15 resulted of modest entity. The impact of DCA treatment on lactate production did not cause significant changes in any of the three OSCC cell lines (Fig. 3B). Consequently, the OxPhos/Glycolysis flux ratio increased largely in HSC-2 and scantly in PE15 with an intermediate value for HSC-3 (Fig. 3C). Importantly, the activity of CIV was not significantly affected by the DCA treatment in any of the three OSCC cell lines (Fig. 3D), as well as the CIV protein content assessed by immunoblotting (not shown).

All together these results show that DCA-treatment promotes a metabolic shift toward oxidative metabolism by enhancing the flux of oxidizable intermediate metabolites. However and most notably, this effect was of different intensity depending on the metabolic profile of the cell tested with those characterized by a prominent glycolytic flux resulting more responsive to the drug.

### Effect of DCA on mitochondrial morphology

**Figure 4:**
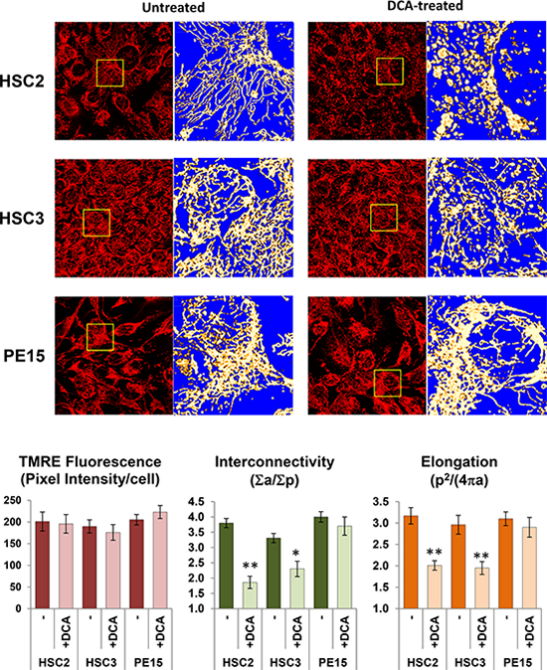
Effect of DCA on mitochondrial morpho-functional parameters The upper panel shows a laser scanning confocal microscopy analysis (LSCM) of the ΔΨ_m_ assessed by the fluorescent probe TMRE in live OSCC cells either untreated and incubated with 4 mM DCA for 24 h. An enlarged detail of the optical field (square) is shown aside of each picture and rendered in false colors to highlight the morphology of the mitochondrial network. Imaging is representative of three different experiments yielding comparable results. The graph bars in the bottom panel show the quantitative analysis of ΔΨ_m_-related TMRE fluorescence and of the morphometric parameters, mitochondrial interconnectivity and elongation, calculated as detailed in Materials and Methods. The data reported are means (± SEM) of values referring to at least ten optical fields randomly selected for each condition and clustered from three independent cell preparations. (*) *P* < 0.05, (**) *P* < 0.01 vs DCA-untreated.

Changes in mitochondrial morphology are emerging as a hallmark for different states of the cell physiology [24]. Using TMRE, we carried out a morpho-functional analysis of the mitochondrial compartment in OSCC cells and the impact on it of DCA (Fig. 4). The results of this analysis showed that the basal level of the ΔΨ_m_-related TMRE fluorescence was comparable among the three OSCC cell lines confirming at the single cell level the observations obtained by flow-cytometry (see Fig. 1C). The morphology of the mitochondrial compartment was characterized by a dense and highly interconnected tubular network with any apparent significant difference among the three OSCC cells lines. Following DCA treatment, the mitochondrial network resulted largely fragmented in HSC-2 cells and, to a lesser extent, in HSC-3 cells but not in PE15 cells. The ΔΨ_m_-related TMRE fluorescence appeared, instead, not significantly affected by DCA treatment in all the OSCC cell samples. Morphometric analysis of the TMRE-related fluorescence signal confirmed in HSC-2 and HSC-3 a significant DCA-mediated decrease of the mitochondrial interconnectivity and elongation.

### Effect of DCA on the cellular redox state

**Figure 5:**
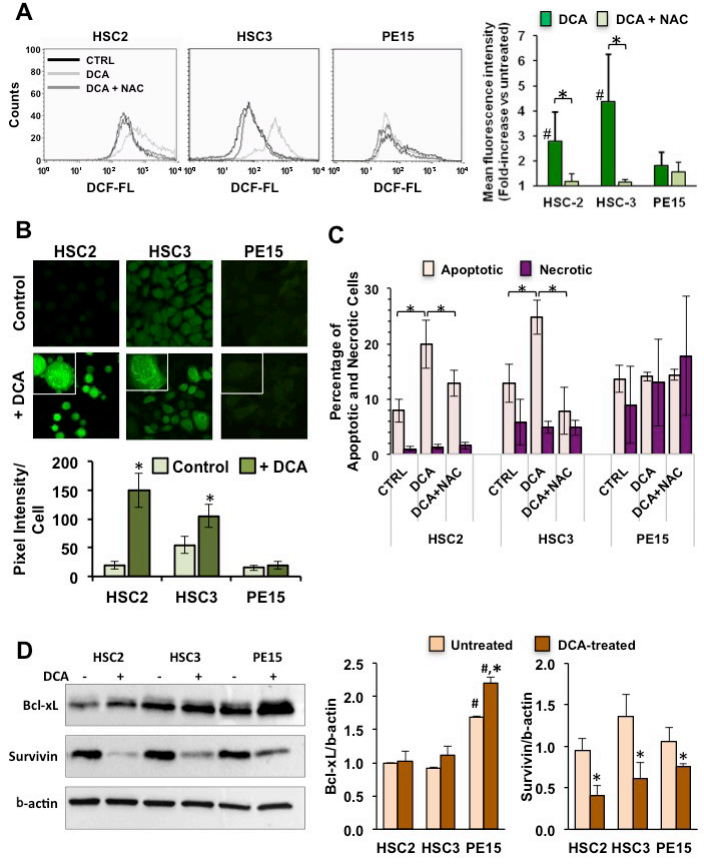
Effect of DCA on intracellular ROS generation and apoptosis **(A)** Cellular ROS production assayed by flow-cytometry fluorescent probe DCF (upper panel). Cells were incubated for 24 h with 10 mM DCA ± 10 mM of the ROS scavenger NAC added 4 h before the analysis. The bar histogram on the right shows the mean intensity of the DCF-related fluorescence (MFI) expressed as fold-change of the untreated cells and are means ± SEM of three independent experiments. (*) *P* < 0.05; (#) < 0.05 vs DCA-untreated. **(B)** Representative LSCM imaging of ROS production in living OSCC cells treated with DCA as in panel (A) and assessed by DCF. Magnification of selected areas (indicated by the white frame) in DCA-treated cells are shown at the top of each panel. The images are representative of three different preparations yielding similar results. The histogram below the images shows the quantitative analysis of the DCF-related fluorescence/cell; the values are means ± SEM of three independent experiments under each condition wherein the digitalized fluorescence images from at least five randomly selected optical fields (each containing about 25 cells) were analyzed. **(C)** Measurement of apoptotic and necrotic cells performed by flow-cytometry after staining cells with annexin-V and propidium iodide. Cells were incubated with 20 mM DCA alone or co-incubated with DCA and 10 mM NAC for 48 h. Data, expressed as percentage of total events analysed, are the means ± SEM of three independent experiments. (*) *P* < 0.05. **(D)** Protein expression levels of the anti-apoptotic factors, Bcl-xL and survivin, assayed by Western blotting in untreated and 10 mM DCA-treated cells for 24 h (left panel); β-actin served as loading control. Graph bars on the right show the average (± SEM) of data resulting from densitometric analysis of three independent blots. (*) *P* < 0.05, (**) *P* < 0.01 vs DCA-untreated; (#) *P* < 0.001 vs HSC-2 and HSC-3.

Data from the literature suggest that the cytotoxic effect of DCA on tumor cells might be caused by unbalance of the cellular redox homeostasis [25]. To verify this possibility we assessed the intracellular reactive oxygen species (ROS) with the peroxide-oxidizable fluorescent probe DCF by flow-cytometry. The results presented in Fig. 5A show that 10 mM DCA treatment for 24 h caused a N-acetyl cysteine (NAC)-sensitive increase of the mean fluorescence intensity in HSC-2 and HSC-3 cell lines (2–3 fold change as compared with the basal ROS level). Conversely, DCA-treatment of the PE15 cell line resulted in a less significant change of the DCF-related fluorescence. These observations were confirmed at the single cell level by confocal microscopy imaging using the same probe (Fig. 5B).

### Effect of DCA on apoptosis

Fragmentation of the mitochondrial network (i.e. enhanced fission) as well as a pro-oxidative setting are considered markers of cell stress, preluding to apoptosis, therefore, we tested whether the cytotoxic effect of DCA was linked to changes in apoptotic markers. Measurement of apoptotic cells was performed by flow-cytometry with the annexin-V/propidium iodide assay (Fig. 5C). Consistent with the MTS assay, 20 mM DCA-treatment for 48 h resulted in an increase in annexin V-positive cells in HSC-2 and HSC-3, reflecting early apoptosis thereof. On the contrary, any appreciable DCA-mediated pro-apoptotic effect was detectable in the PE15 cells. HEK 293 cells displayed a negligible change of the apoptotic marker following identical DCA treatment (data not shown). Interestingly, the amount of annexin V-propidium iodine double stained cells in HSC-3 and PE15 was somehow higher than in HSC-2, reflecting necrosis or later stage apoptosis. However, no difference in the percentage of necrotic cells was observed following DCA treatment.

Further we assessed the protein expression level of two anti-apoptotic factors, Bcl-xL and survivin, by Western blotting. As shown in Fig. 5D, the basal level of Bcl-xL was significantly different among the three OSCC cells with the PE15 exhibiting about 70% higher amount than HSC-2 and HSC-3. Unlike Bcl-xL, the survivin content was comparable in all the three OSCC cells. Most notably, DCA treatment resulted in no significant change in the level of Bcl-xL in HSC-2 and HSC-3 and in a slight increase in PE15 whereas it caused a significant decrease of the survivin content in all the OSCC cells, which was larger in both HSC-2 and HSC-3 (−50%) than in PE15 (−25%).

Finally, to verify whether the DCA-induced enhanced apoptosis was linked to the pro-oxidative state elicited by the compound, OSCC cells were co-treated with DCA and NAC, the latter under conditions proved to prevent the DCA-induced overproduction of ROS (Fig. 5A). Figure 5C shows that NAC largely prevented or suppressed the DCA-mediated apoptosis in HSC-2 and HSC-3 respectively. Conversely, NAC did not cause significant changes neither of apoptotic nor of necrotic PE15 cells.

## DISCUSSION

OSCC is the most frequent carcinoma of the oral cavity [26] with poor survival due to its refractariety to conventional therapy, thereby search of new pharmacological approaches is pressing [27]. Over the last few years, targeting of mitochondria has become an attractive strategy in cancer chemotherapy [28, 29]. The underlying rationale is based on the belief that reprogramming cancer cell metabolism toward an aerobic phenotype might cause, in the contest of mitochondria dysfunctions, a bioenergetic collapse promoting cell death. However, it has to be considered that not all the cancer cells share the “Warburg phenotype” [30]. Indeed several exceptions have been described [31] and a reassessment of the mitochondrial metabolism in the cancer cell physiology is under evaluation [32]. Nevertheless, pinpointing cancer subsets, specifically vulnerable to mitochondria-targeted therapy, is a worthy challenge.

In the present study we characterized the mitochondrial phenotype of three different OSCC-derived cell lines and assessed their biological response to the metabolic drug DCA.

In our investigation, the measurement of the mitochondrial respiratory activity in intact OSCC cells underscored the metabolic heterogeneity occurring when dealing with different tumour cell lines. Indeed, while the HSC-2 and HSC-3 displayed a relatively low mitochondria-mediated OCR, the PE15 exhibited a two-fold higher value. The overall activity of respiratory chain is kinetically controlled by complex IV depending on the membrane energy state [22]. Consistently, the specific activity and protein content of complex IV was significantly and specifically higher in the PE15 cells. However, the catalytic activity of the complex has been proved to be tuneable by post-translational covalent modifications as well as by interactions with regulatory factors [33]. The occurrence of differential modulatory pathways controlling the activity of the respiratory chain complexes in specific cancer cell types warrants further investigations.

Importantly, the OCR sensitive to oligomycin, and therefore involved in energy conversion, confirmed for PE15 the highest OxPhos efficiency. Measurement of the lactate flux released in the cell culture medium resulted in significantly larger values for HSC-2 and HSC-3 cells as compared with PE15, confirming more reliance on glycolysis thereof.

The lower OxPhos efficiency in HSC-2 and HSC-3 cells cannot be attributed to a different content of mitochondria or expression of the OxPhos complexes. One possible explanation for the attenuated OxPhos efficiency in HSC-2 and HSC-3 might stem in the relatively larger insensitivity of the OCR to oligomycin, which is considered a measure of ΔΨ_m_-dissipating activities (i.e. proton leak) that lower the conversion of the oxidative energy in ATP synthesis. Another plausible explanation might rely on the differential expression of mitochondrial carriers controlling the influx of respiratory metabolites/substrates. Moreover, we cannot exclude that the mutated p53 gene in HSC-2 and HSC-3, could account for their less efficient OxPhos activity. p53 plays a pivotal role in the regulation of energy-generating metabolic pathways and its loss induces dramatic metabolic alterations toward a glycolitic phenotype [34]. In particular, it has been demonstrated that p53 directly regulates the synthesis of cytochrome c oxidase 2 (SCO2), required for the assembly of the cytochrome c oxidase complex [35]. Further studies will shed lights on these issues.

Taken all together and in keeping the relatively low efficiency of glycolysis in ATP production as compared with the OxPhos system, our observations denote for HSC-2 and HSC-3 cells a metabolic phenotype with a poor bioenergetic competence.

DCA is a generic low-price drug, in use in humans for more than 30 years, whose molecular mechanism of action remains largely elusive. Impressive results in a wide spectrum of human cancers cells including pancreatic, colon, breast ovarian, endometrial, neuroblastoma and glioblastoma cells were reported for DCA treatment [11–18]. Recently, additive effects were also observed in experimental studies with DCA used in combination with radiation or other drugs [36, 37] and clinical trials are currently testing the DCA anti-tumor effects on different human cancers [38]. However, to the best of our knowledge this is the first study addressed to test the effect of the drug on oral squamous cells carcinomas with the aim to correlate them with the cellular metabolic phenotype. The results here presented clearly show that under conditions promoting comparable significant dephosphorylation of the E1-α subunit of PDH in the three different OSCC cell types, DCA treatment caused a distinctive and dramatic cytotoxic effect only on HSC-2 and HSC-3 whereas scarcely affected cell viability in PE15 as well as in a non-tumoral cell line. Most notably the mitochondria-related oxidative metabolism was apparently up-regulated following DCA-treatment to a significant extent only in HSC-2 and HSC-3 cell lines with a minor if any effect on the lactate production. As a consequence DCA was able to elicit a major shift in the balance between the glycolytic vs respiratory metabolism in HSC-2 and HSC-3 leaving almost unchanged that of the PE15 cell line. Although the cytotoxic activity of DCA has been attributed to PDK inhibition in solid tumor cell models [19], the reported effective concentrations of DCA *in vitro* (also used in this study) are several log greater than the inhibition constant (Ki) of PDK, thereby implying additional effects.

Our observations suggest that DCA treatment causes an unbalance in the redox homeostasis, leading to overproduction of reactive species. This is accompanied with a profound de-structuration of the mitochondrial network in HSC-2 and HSC-3 resulting in an extensive fragmentation likely derived from deregulation of the organelle fusion/fission machinery [39, 40]. Accordingly, preliminary results obtained by our group indicate a DCA-mediated change in the expression of factors known to be involved in the mitochondrial dynamics (i.e. up-regulation of Drp1, down-regulation of OPA1 and Mfn1; manuscript in preparation). Fragmentation of mitochondria is considered a step preluding to processing of damaged organelles by mitophagy, a specialized form of macro-autophagy [41]. When removal of dysfunctioning mitochondria overcomes the cell capacity for “quality control”, the apoptotic program is activated. Consistently, we found in HSC-2 and HSC-3 evidences of DCA-induced proapoptotic priming.

**Figure 6:**
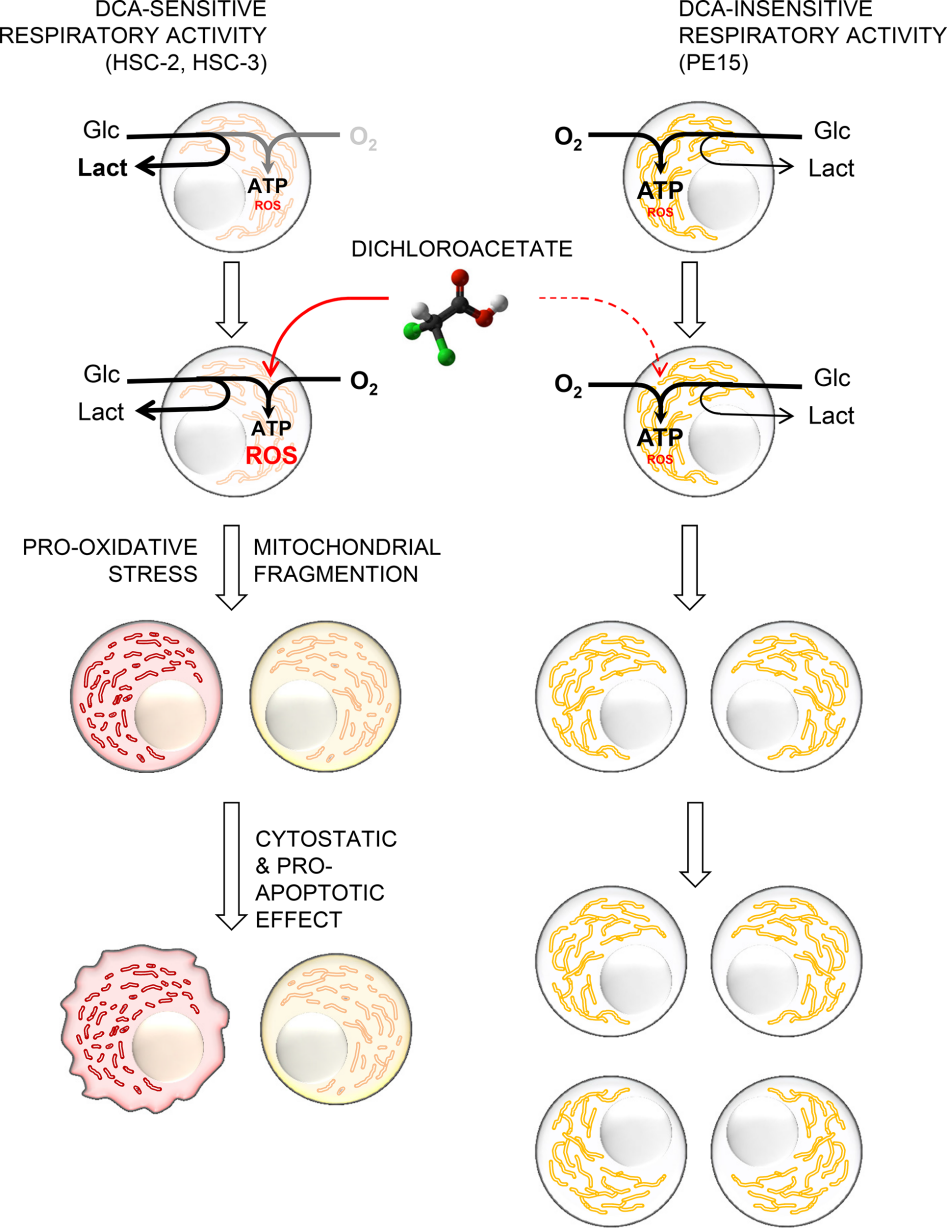
Proposed mechanism of the differential effect of DCA on OSCC-derived cell lines The proposed model schematically illustrates how the metabolic profile can affect the cellular response to DCA and determine its cytotoxic potential on OSCC cells. HSC-2 and HSC-3 cell lines, adapted to a relatively low oxidative metabolism and high glycolysis displayed a DCA-sensitive respiratory activity. This metabolic feature may predispose cells to a major sensitivity to DCA-induced apoptosis, associated to oxidative stress and mitochondrial fragmentation. On the contrary, PE15 cell line, in which the more efficient respiratory activity resulted to be DCA-insensitive, displays an intrinsic resistance to the detrimental DCA-induced effects.

It is tempting to speculate that unlike PE15, HSC-2 and HSC-3 being adapted or programmed to a low-regimen oxidative metabolism are not suited to cope with an enhanced O_2_ metabolism. This might depend on subtle dysfunctions in mitochondria, which, if tolerated under conditions of low cell activity, become detrimental once the cell is forced to rely on them for the bioenergetic demand (see the schematic picture in Fig. 6).

In conclusion, our study demonstrates for the first time the efficacy of DCA in producing remarkable cytotoxic effect in cells derived from human oral carcinomas. This effect appears to inversely correlate with the mitochondrial respiratory capacity of the tumour cells and provides a rationale to reconcile the heterogeneous and sometimes conflicting outcomes of the reported DCA-effects *in vitro*. Further experiments focusing on *in vivo* models will be needed to confirm the selective cytotoxic effect of DCA on the tumor mass.

The easy accessibility of the oral cancers enables, by a simple non-invasive biopsy, to isolate and test primary tumour cells for their responsiveness to cytotoxic assays. In case of positive outcome a local/topical administration of DCA could be provided, thus limiting the toxic effects of systemic treatment. This approach might benefit of a newly developed potential drug, which conjugates by a biodegradable linker DCA to the lipophilic triphenylphosphonium cation allowing selective delivery to mitochondria at locally high concentration [42]. This innovative therapeutic strategy, associated to a predictive test, might enable to develop new personalized treatment for oral cancers.

## MATERIALS AND METHODS

### Reagents and cell culture

Reagents were purchased from Sigma-Aldrich (St. Louis, MO) unless otherwise specified. Human OSCC cell lines, were maintained at sub-confluent conditions in RPMI 1640 (HSC-2 and HSC-3) and DMEM (PE15) media, supplemented with 10% fetal bovine serum, L-glutamine (2 mM) and penicillin–streptomycin (100 U/ml) at 37°C in a 5% CO_2_ humidified atmosphere.

### Measurement of endogenous respiration rates in intact cells

Cultured cells were gently detached from the dish by trypsinization, washed in PBS, harvested by centrifugation, and immediately assessed for O_2_ consumption by a Clark-type electrode (Hansatech) in a thermostated gas-tight chamber equipped with a stirring device. Five-seven x10^6^ viable cells/ml were assayed in 50 mM KPi, 10 mM Hepes, 1 mM EDTA (pH 7.4) at 37°C; after attainment of a stationary endogenous substrate-sustained respiratory rate, 1 μg/ml of oligomycin and 400 nM carbonylcyanide-p-trifluoromethoxyphenylhydrazone (FCCP) were added sequentially within a 10-minute interval. The rates of O_2_ consumption (OCRs) were corrected for 1 μM rotenone-insensitive respiration. The respiratory control ratio (RCR) was obtained by dividing the rate of oxygen consumption achieved before the addition of oligomycin by the rate after the addition of oligomycin. The OCRs were normalized to the cell number or to the cellular protein concentration. The latter was determined by the Bradford assay (Bio-Rad).

### Measurement of NAD^+^ and NADH

Cells, collected by trypsinization and centrifugation, after protein determination, were suspended in 15% HClO_4_ (NAD^+^ extraction) or 0.2 M NaOH (NADH extraction). After 10 min, the suspensions were neutralized by adding 0.1 M KOH (NAD^+^ extraction) or 0.1 M HCl (NADH extraction). After centrifugation, the supernatants were collected and assayed immediately. NAD or NADH concentrations were measured by a cyclic enzyme reaction system in which alcohol dehydrogenase reduces dichlorophenol indolphenol (DCIPIP) through the intermediation of phenazine methosulfate (PMS). The reaction mixture consisted of 0.63 ml of 100 mM phosphate Buffer (pH 7.5), 0.03 ml of 30 mM phenazine methosulphate (PMS), 0.04 ml of 0.6 mM dichlorophenol indolphenol (DCIPIP), 0.1 ml of 95% ethanol, 5 units ADH. The reaction was started by the addition of 200-300 μg of the sample. Reduction of the blue-colored DCPIP to colorless DCPIPH_2_ was measured by recording the decrease in absorbance at 600 nm. The concentration of NADH and NAD^+^ in each extract was determined by comparing sample values to standard curves generated from samples containing known amounts of NADH and NAD^+^ that had been cycled under identical conditions as the samples.

### Lactate measurements

A total of 2 × 10^6^ cells were treated for 24 h with 10 mM DCA and lactate was measured in culture medium using a Lactate Colorimetric Assay Kit (Abcam, Cambridge, MA, USA). Data were normalized to total protein amounts in each sample.

### Flow cytometric detection of mitochondrial membrane potential and ROS

To measure mitochondrial membrane potential (ΔΨ_m_) and ROS, cells were incubated, protected from light, with 1 μM tetramethylrhodamine ethyl ester (TMRE) or with 2 μM 2,7-dichlorofluorescin diacetate (DCFH-DA) (Molecular Probes, Eugene, OR), at 37°C for 15 min and analyzed by a FACScantoII flow cytometer, (Becton Dickinson). The emitted fluorescent signal of 10,000 events for each sample was acquired and analyzed with the CellQuest software.

### Mitochondrial respiratory complexes activity

The specific activities of NADH: ubiquinone oxidoreductase (complex I) and cytochrome c oxidase (complex IV) were assayed spectrophotometrically on frozen–thawn and ultrasound-treated cells in 10 mM Tris, 1 mg/ml serum albumin, pH 8.0. Complex I was assayed (in the presence of 1 μg/ml of antymicin A plus 2 mM KCN) by following the initial 2 μg/ml rotenone-sensitive rate of 50 μM NADH oxidation (ε340 nm = 6.22 mM^−1^ cm^−1^) in the presence of 200 μM decylubiquinone (dUQ) as electron acceptor. CIV activity was assayed by following (in the presence of antimycin A) the initial KCN-sensitive rate of ferro-cytochrome c oxidation (ΔA at 550 nm, ε = 21.1 mM^−1^cm^−1^) under aerobic conditions.

### Western blotting analysis

Cultured cells were lysed in 150 mM NaCl, 0,1% SDS, 5 mM EDTA, 1% sodium deoxycholate, 1% Triton X-100, 50 mM Tris, pH 7.4 and protease inhibitor cocktail and centrifuged at 12,000 g for 15 min at 4°C to remove cellular debris. The protein concentration was measured using the Bradford assay (Bio-Rad). The proteins were separated by 12% SDS polyacrylamide gel electrophoresis and transferred to a PVDF membrane (Bio-Rad). After blocking with 5% skim milk for 1 hour, the membranes were incubated with MitoProfile Total OXPHOS Human WB Antibody cocktail (1:500; Abcam Cambridge, UK), PDH-E1α (1:2,000; Abcam, Cambridge, UK), phospho-PDH-E1α (1:500; Abcam Cambridge, UK), Bcl-xl (1:1,000; Cell Signaling Technology), survivin (1:1,000; Cell Signaling Technology), GAPDH (1:2,500; Santa Cruz Biotech) and β-actin (1:5,000; SIGMA), overnight at 4°C. After incubation with corresponding suited 1:2.500 horseradish peroxidase-conjugated secondary antibody (1,2500; Cell Signaling Technology); the signals were developed using the enhanced chemiluminescence kit (ClarityTM Western ECL Substrate, Bio-Rad) and the ChemiDoc Imaging System XRS + (BioRad) and analysed with the Image Lab 4.1 software.

### Cell proliferation assays

Cell proliferation was assessed by xCELLigence RTCA MP System (Roche, Germany) that monitors cellular events in real time by measuring impedance-based signals [43]. Cell-sensor impedance is expressed as Cell Index (CI). Experimental results were performed using RTCA Software 1.2 that calculated the population doubling by fitting the curve to an exponential equation.

### Cell viability assays

Cells were seeded in a 96-well culture plate and after 24 h of incubation with DCA, cell viability was measured using solutions of a novel tetrazolium compound (3-(4,5-dimethylthiazol-2-yl)-5-(3-carboxymethoxyphenyl)-2-(4-sulfophenyl)-2H-tetrazolium, inner salt; (CellTiter 96^®^ AQueous MTS Reagent Powder, Promega) and the electron coupling reagent, phenazine methosulfate, PMS. MTS is bioreduced by cells into a formazan product that is soluble in tissue culture medium. The absorbance of the formazan at 490 nm was measured directly from 96-well assay plates using the Plate Reader (das srl, Italy) with a reference filter at 630 nm. Three independent measurements were performed in triplicate for each assay.

### Live cell imaging of ROS and mtΔΨ

Cells cultured at low density on fibronectin-coated 35-mm glass-bottom dishes were incubated for 20 minutes at 37°C with 2 μM TMRE or 10 μM DCFH-DA (Molecular Probes, Eugene, OR) to monitor mtΔΨ and ROS respectively. Stained cells were washed with PBS and examined by a Nikon TE 2000 microscope (images collected using a 60X objective [1.4 NA]) coupled to a Radiance 2100 dual-laser (4-line Argon-Krypton, single-line Helium-Neon) confocal laser scanning microscopy system (Biorad). Acquisition, storage, and analysis of data were performed with LaserSharp and LaserPix software from Biorad or ImageJ1.48u (Wayne Rasband, NIH, USA, http://imagej.nih.gov/ij).

### Apoptosis assay

Apoptosis was detected by flow cytometry (FACScantoII, Becton Dickinson) following staining of cells for Annexin-V-FITC and PI (BD Biosciences), after 48 h of incubation with DCA (20 mM). Three independent experiments were carried out. Ten thousand events were collected per sample.

### Statistical analysis

The unpaired Student's t-test and one-way ANOVA followed by Newman–Keuls tests were used to compare continuous variables. All the data are expressed as the mean ± standard error mean (SEM); a *P* value <0.05 was accepted as statistically significant.
